# Clinical characteristics of depressed children and adolescents with and without suicidal thoughts and behavior: a cross-sectional study

**DOI:** 10.3389/frcha.2025.1510961

**Published:** 2025-02-21

**Authors:** I. Häberling, M. Preisig, S. Emery, N. Baumgartner, M. Albermann, M. Strumberger, K. Schmeck, L. Wöckel, S. Erb, B. Rhiner, B. Contin, S. Walitza, G. Berger

**Affiliations:** ^1^ Clinic for Child and Adolescent Psychiatry and Psychotherapy, Psychiatric University Hospital Zurich, Zurich, Switzerland; ^2^ Outpatient Psychology Wil, Psychiatric Hospital St. Gallen Nord, Wil, Switzerland; ^3^ Outpatient Clinic, Psychiatric Services Lucerne, Lucerne, Switzerland; ^4^ Department of Clinical Research, University of Basel, Basel, Switzerland; ^5^ Center for Child and Adolescent Psychiatry and Psychotherapy, Clienia Littenheid AG, Littenheid, Switzerland; ^6^ Special Offers and Projects, Child and Adolescent Services St. Gallen, St. Gallen, Switzerland; ^7^ Child and Adolescents Psychiatry, Psychiatric Services Thurgau, Weinfelden, Switzerland; ^8^ Child and Adolescent Psychiatry, Psychiatric Services Baselland, Liestal, Switzerland

**Keywords:** depression, suicide attempts, suicidal ideation, children, adolescents, non-suicidal self-injury

## Abstract

**Introduction:**

About half of all adolescents with major depressive disorder (MDD) have frequent suicidal thoughts and of those with suicidal ideations, about one-third attempt suicide. Identifying clinical characteristics associated with suicidal ideation and attempts is important for suicide prevention and clinical care.

**Methods:**

Participants were four groups of adolescents diagnosed with MDD (*n* = 246, 180 females): (a) non-suicidal youths (*n* = 76), (b) ideators (*n* = 102; current suicidal ideation), (c) ideator-attempters (*n* = 56; current suicidal ideation and lifetime history of suicide attempt), and (d) lifetime attempters (*n* = 12; no current suicidal ideation but lifetime history of suicide attempt). Adolescents underwent clinical interviews and completed questionnaires assessing sociodemographic and clinical variables. Multivariate analyses of variance, logistic regression models, mediation and moderation analyses were run to assess which variables were associated with group membership.

**Results:**

Suicidal ideators, irrespective of whether they had attempted suicide previously, had higher depression severity, higher anxiety and lower resilience compared to non-ideators. Hopelessness was associated with greater odds of being a suicidal ideator (*p* < .001, *OR* = 1.18) or an ideator-attempter (*p* = 0.036, *OR* = 1.13) than a non-suicidal youth. Attempter-ideators engaged more often in self-harm behavior compared to ideators (*p* = 0.046, *OR* = 1.13) and non-suicidal youths (*p* < .001, *OR* = 1.45). Ideator-attempters had experienced more childhood maltreatment, with hopelessness mediating the relationship between childhood maltreatment and suicidal ideation. Self-harm moderated the relationship between suicidal ideation and the probability of having made a suicide attempt.

**Limitations:**

Only cross-sectional data was included, and data was based mostly on self-report measures.

**Conclusions:**

Suicidal thoughts are associated with increases in hopelessness while suicide attempts are linked to non-suicidal self-harm behavior. Treatment of non-suicidal self-harm behavior might be an effective suicide prevention strategy in young people with depression.

**Clinical Trial Registration:**

www.ClinicalTrials.gov, identifier (NCT03167307).

## Introduction

Adolescence is a critical period for suicidal behavior, especially in those suffering from major depressive disorders (MDD). While 12%–29.9% of all youths experience suicidal ideation at some point in their life (1–3), approximately 38%–50% of adolescents with MDD report recurrent suicidal ideations (4, 5). Moreover, while the lifetime prevalence for suicide attempts in adolescents is estimated to lie between 4.1% and 16.9% globally (1–3), one in five depressed adolescents will attempt suicide by the age of 30 (6). In the general adolescent population, suicide is the fourth leading cause of death globally (7–9) and adolescents suffering from MDD have a six- to eightfold increased odd of attempting suicide (2, 6), with an even higher risk among those suffering from additional comorbidities (10). More than half of adolescents who die by suicide suffered from depression (11, 12). Even among patients with MDD, adolescents have the highest risk of suicide attempts compared to children, young adults or adult MDD patients (6, 8). Earlier onset of MDD is associated with more frequent suicidal thoughts and attempts (13, 14), and since a suicide attempt is one of the main risk factors for death by suicide (15), adolescents with MDD are at high risk for future suicidal behavior.

Although suicidality is one of the diagnostic criteria for MDD, some depressed adolescents do not develop any suicidal thoughts or behaviors. The clinical characteristics of depressed adolescents with and without suicidality have been reported to be quite similar so far (16), although the severity of clinical symptoms seems to be higher overall in suicidal depressed adolescents (17). Barbe et al. (18) compared clinical characteristics of 43 suicidal depressed adolescents with 92 non-suicidal depressed ones, and found that suicidal youths were more severely depressed, more often hopeless, had more severe functional impairments, and suffered more often from insomnia. Similarly, Liu et al. (19) found suicidal children and adolescents to be more severely depressed, to have more depressive symptoms, and to have more comorbid disorders. In addition, the suicidal group felt worthless more often and had more feelings of guilt than the non-suicidal youths. A recent systematic review and meta-analysis by Ezquerra et al. (17) further corroborates these findings, revealing that children and adolescents who have attempted suicide multiple times exhibit significantly higher levels of depression, anxiety, substance abuse, aggression, and hopelessness compared to those who have made a single suicide attempt.

Suicidal and non-suicidal depressed adolescents may have similar clinical symptoms, as known risk factors for suicidality, such as adverse childhood experiences and certain personality traits, are also risk factors for the development of a depressive episode itself (20). Sexual, emotional and physical abuse and neglect in childhood increase the likelihood of developing depression and suicidality in adulthood (21–23), with the risk for suicidal behavior increasing when more than one type of maltreatment is present (24). Furthermore, maltreatment occurring between the ages of six and 13 is associated with the highest risk of developing depression in adulthood, compared to maltreatment experienced in early childhood or late adolescence (21). About half of adult MDD patients report adverse childhood experiences, and these patients not only have more severe depression, but are more likely to have suicidal thoughts and attempts (25), and suffer more chronic forms with poorer treatment outcomes (26). Childhood maltreatment experiences have also been associated with suicidal behavior in a general population of adolescents (27) and in adolescent mood disorder patients (28, 29). Chen et al. (28, 29) found that childhood maltreatment experiences were associated with a slightly increased odd of suicide attempts (OR = 1.04) in adolescents experiencing their first depressive episode.

According to the hopelessness theory of depression and suicidality (30, 31), stressful life events, such as experiences of abuse in childhood, promote the development of a negative cognitive style. When an individual attributes negative events to stable and global causes, adverse situations are seen as unchangeable. As a result, feelings of hopelessness arise and suicide seems to be the only solution to end an unbearable condition. In a longitudinal study on 249 adolescents who were followed up over a 2.5-year period, hopelessness predicted the first onset of a depressive episode, as suggested by the hopelessness theory of depression, although in that study, suicidality was not specifically assessed (32). Furthermore, hopelessness has been shown to be one of the most important predictors of suicidality (33, 34). For example, in a population-based study of over 70,000 students in the US, hopelessness and depressive symptoms were identified as the main factors distinguishing youths with suicidal behavior from those without (35). Further, a systematic review (36) of 18 studies with young adults aged 18–30 years identified hopelessness an important predictor of suicide.

According to Nock et al. (2), about one third of depressed adolescents with frequent suicidal ideations will attempt suicide, whereas a more recent study (37) and a meta-analysis (38) found lower percentages of 8.7% and 17% of suicidal adolescents to attempt suicide, respectively. So far it has been difficult to find factors associated with the transition from ideations to attempts (15). In a study comparing depressed adolescent ideators and attempters, anhedonia was found to be the only depressive symptom that differed between the two groups, with suicide attempters suffering from greater anhedonia (39). A study involving 7,491 adolescents found that those who experienced sadness, hopelessness, sexual violence, and substance abuse had higher odds of engaging in suicidal behavior, rather than merely experiencing suicidal ideation (40). In a separate study, Florez et al. (41) identified several factors that best differentiated suicide attempters from ideators in a sample of 580 adolescents aged 10–17 years, including female gender, older age, number of inpatient hospitalizations, legal issues, and higher impulsivity.

According to Joiner's interpersonal-psychological theory of suicide [IPTS; (42)], a suicide attempt only occurs when the desire to die is combined with the capability to act on that desire. The acquired capability for suicide is developed through experiences of pain, such as experiences of abuse in childhood, drug use, previous suicide attempts or non-suicidal self-injury (NSSI). In adolescents, studies of NSSI appear to confirm the predictions of the IPTS. In a large study that included four different samples, namely adolescent psychiatric patients, adolescent high school students, university undergraduates and random samples of adults, the only variables consistently associated with suicide attempts were suicidal ideations and NSSI (43). In two large clinical trials on depressed adolescents, the Adolescent Depression Antidepressants and Psychotherapy Trial [ADAPT; (44)] and the Treatment of Resistant Depression in Adolescents Trial [TORDIA; (45)], NSSI at baseline predicted suicide attempts over a 24 and 28 months follow-up period, respectively. A review (46) of 17 studies found that greater NSSI frequency predicts suicide attempts and NSSI typically precedes a suicide attempt. In the Minnesota Student Survey, which included over 70,000 students, NSSI was the most important factor distinguishing between youths who think of suicide and those who attempt it (35), a finding which is also supported by an up to date systematic review by Kirshenbaum et al. (33). Despite these findings, a meta-analysis concluded that the prediction of suicide attempts by previous self-harming thoughts and behaviors is still weak, and only slightly above chance (47).

In the current study, it was examined whether known risk factors for depression and suicidality differentiate between depressed adolescents with (a) no suicidal ideation (SI) and no history of suicide attempts (SA); (b) current ideation but no lifetime attempts; (c) current ideation and lifetime attempts, and (d) lifetime attempts but no current suicidal ideation. A large sample of 246 children and adolescents aged 8–18 years with diagnosed MDD of at least moderate severity was included. According to the literature reviewed, it was hypothesized that suicidal and non-suicidal youths will have similar clinical features, but the suicidal ones would have a higher overall severity. Following the hopelessness theory on suicide and depression (30, 31), it was tested whether hopelessness mediates the relationship between childhood maltreatment and suicidal ideation. In addition, based on the IPTS (42), it was hypothesized that suicide attempters, compared to individuals who solely think about suicide, are more likely to engage in self-harming behavior, are more likely to use drugs, and are more likely to have experienced childhood maltreatment.

## Methods

### Procedure

Patients were recruited as part of the clinical trial “Omega-3 fatty acids as a treatment for pediatric depression” (48). Inclusion criteria for the trial was a diagnosis of MDD according to DSM-IV (49) of at least moderate severity with a score of ≥40 on the *Children's Depression Rating Scale Revised* [CDRS-R; (50)]. Exclusion criteria were a lifetime diagnosis of schizophrenic disorder, affective bipolar disorder, substance use disorder or a diagnosis of an eating disorder within the last six months. In contrast to other clinical trials on depression (51), acute suicidality was not an exclusion criterion. Further inclusion and exclusion criteria are listed in the clinical trial design paper (48). This study only includes data collected during screening visits prior to randomization to a treatment arm. Assessments were carried out in-person, either in the participating centers or at the participants' homes. All procedures were approved by the local ethics committees and the clinical trial was registered at clinical.trials.gov (NCT03167307).

### Sample

A total of 310 children and adolescents were screened for inclusion into the omega-3 depression trial. 257 children and adolescents met all inclusion criteria and were randomly assigned to one of the treatment arms. As it was the main variable of interest, 11 patients had to be excluded from the current analyses due to missing suicidal ideation scores, resulting in 246 children and adolescents aged 8–18 years with diagnosed major depressive disorder. All patients were divided into four groups based on their reported suicidal thoughts and behaviors: (a) non-suicidal youths (NO; *n* = 76, 30.9%) showed low scores (<31) on the suicidal ideation questionnaire (SIQ) (52) and no history of suicide attempts, (b) suicide ideators (ID; *n* = 102, 41.5%) had elevated scores (≥31) on the suicidal ideation questionnaire (SIQ) but no lifetime history of suicide attempts, (c) ideator-attempters (AT+; *n* = 56, 22.8%) had elevated scores (≥31) on the SIQ and a lifetime history of suicide attempts, and (d) lifetime attempters (AT−; *n* = 12, 4.9%) who had a lifetime history of suicide attempts but no current suicidal ideation (SIQ < 31).

### Interview and questionnaires

The German version of the *Children's Depression Rating Scale—Revised* (CDRS-R; (53), a semi-structured clinical interview initially developed by Poznanski et al. (50), was used to assess the severity of depression in children and adolescents. The interviewer asks parents and children to rate the severity of 14 depression-related symptoms, and combines the two reports into one composite score. Three additional non-verbal symptoms are rated by the interviewer based on behavioral observations of the child interviewed. All the symptom scores are then added to reach a final score, indicating depression severity. As the scale also includes two items regarding suicidality (suicidal ideation; morbid ideation), the total score was corrected for these two items and this new score was used for determining depression severity (CDRS-S). The scale shows acceptable internal consistency in the present sample (Cronbach's *α* = 0.64).

The *Childhood Trauma Questionnaire (CTQ)* is a self-report instrument used to assess experiences of childhood maltreatment (54, 55) by indicating whether family members engaged in a certain set of behaviors while the participant was growing up. These behaviors include situations in which the child was sexually, physically or emotionally abused, for example by being molested, hit or belittled. Furthermore, items regarding emotional and physical neglect are also included. A total maltreatment score is built by adding the scores of all subscales. In addition, the authors provide cut-off scores for assessing the severity of each type of maltreatment. As in earlier research, an abuse type was rated as present when it reached at least moderate severity (emotional abuse ≥13; physical abuse ≥10; sexual abuse ≥8; emotional neglect ≥15; physical neglect ≥10). The CTQ is a widely used screening instrument for childhood maltreatment, with good psychometric properties for the German version (56) and also in adolescents (57). The data of this study confirms good internal consistency of the CTQ (Cronbach's *α* = 0.89).

The *Suicidal Ideation Questionnaire-Junior (SIQ-Jr)* was developed to assess suicidal thoughts in children in grades seven to nine (58), but it can also be administered in younger children and adolescents (52). The short version used in the current study consists of 15 suicide-related thoughts with the youths rating whether, and if so, how often they experienced a particular thought in the past month. The scores of the 15 items are then added to build a final score, with a value of 31 or higher indicating a significant level of suicidal ideation (52). The scale exhibited excellent internal consistency in the sample of this study (Cronbach's *α* = 0.95).

The *Beck Hopelessness Scale (BHS)* consists of 20 items either rated true or false, with higher scores indicating more hopelessness (59). Higher scores on the BHS have been shown to be associated with suicidal behavior (60). It may be administered in adolescents aged 13 (61), and it has adequate psychometric properties in German (62), which is corroborated by the data of this study (Cronbach's *α* = 0.89).

The *Beck Anxiety Inventory (BAI)* consists of 21 items rated on a four point Likert scale, asking about symptoms of anxiety during the last week (63). The scores are added, with a higher total score indicating a higher level of anxiety. The scale is suitable for adolescents and there is a German version with good psychometric properties (64). In this study, the BAI yields excellent internal consistency (Cronbach's *α* = 0.92).

The *Connor-Davidson-Resilience Scale (CD-RISC)* is a widely used scale to measure resilience (65), which has been translated into German (66). The version used here consists of 25 items, asking how participants dealt with certain situations during the past month, for example whether they could confront difficult situations with humor. The scale shows excellent internal consistency in this sample (Cronbach's *α* = 0.91).

NSSI was assessed using four items of the *Scale of Impulsivity and Emotion Dysregulation* [IES-27; (67)]. Patients were asked whether, and if so, how often (never, 1–2 times, 3–10 times, daily, several times per day) they harmed themselves during the past month using four different methods (self-battering, burning, deep cutting, cutting/scratching). Scores are added to estimate the frequency of NSSI during the past month. The scale shows high internal consistency in the present data (Cronbach's *α* = 0.93).

Substance use was assessed with the *Alcohol, Smoking and Substance Involvement Screening Test (ASSIST)*, version 3, which has been developed by the World Health Organization (68). Patients are asked whether they had tried eight different classes of drugs for recreational purposes, which are, when affirmed, followed by additional questions regarding consumption patterns. The *lifetime substance use score* is the sum of drug classes that have been used in lifetime, while the *global continuum of substance risk score* additionally integrates the frequency of the drug use. In the present sample, the scale shows acceptable internal consistency (Cronbach's *α* = 0.64).

*Information on sociodemographic information and treatment and illness history* was collected by perusing the clinical records of the patients. In case of missing information, either the parent or the adolescent was asked to provide the missing information.

### Data analytic overview

First, a series of univariate analyses to identify demographic and clinical factors that differentiated between the predefined groups were conducted. For categorical variables, Chi-square statistics were used and for continuous variables one-way analysis of variance (ANOVA). Welch Test was used when homogeneity of variances was violated. In cases of non-normal distribution of the continuous variable, Kruskal–Wallis Test was applied. Analyses of clinical characteristics were additionally adjusted for gender and depression severity. The clinical symptom pattern was analyzed using multivariate analyses of variance on all CDRS-S symptoms with group membership as factor, adjusted for gender. For all analyses, Bonferroni-corrected pairwise comparisons were used to test for group differences.

All measures that differentiated between groups were then entered into an omnibus logistic regression model to predict group membership. Multicollinearity was assessed using Pearson correlation coefficients (see Supplementary Table 1 for the correlation matrix). Collinearity was defined as a Pearson correlation coefficient of *|r*| > 0.5 (69). Acknowledging that thresholds for detecting collinearity can vary and are sometimes set higher (e.g., |*r*| > 0.7), three models were performed. The first model included all predictors, the second model included hopelessness but excluded resilience, and the third model included resilience but excluded hopelessness. This approach was taken due to the relatively high correlation between hopelessness and resilience (*r* = −0.695). In the full model including both hopelessness and resilience, the only predictors that reached statistical significance were hopelessness [*χ*²(3) = 9.98, *p* = .019] and self-harm [*χ*²(3) = 15.72, *p* = .001]. Resilience did not reach statistical significance in the full model [*χ*²(3) = 4.13, *p* = .248], whereas it was statistically significant in the model excluding hopelessness [*χ*²(3) = 14.96, *p* = .002]. This implies that hopelessness may be a more important predictor than resilience, which is why the model including hopelessness but excluding resilience is reported in the main manuscript (see Table 1). For completeness, the other models are reported in the supplementary information (see Supplementary Tables 2, 3).

**Table 1:** Multinomial logistic regression of variables associated with the groups based on suicidal ideation and behavior.

	NO vs. ID		NO vs. AT+		NO vs. AT−		ID vs. AT+		ID vs. AT−		AT + vs. AT−	
	OR	95% CI	OR	95% CI	OR	95% CI	OR	95% CI	OR	95% CI	OR	95% CI
BAI	1.01	0.97–1.05	1.02	0.98–1.07	0.98	0.90–1.08	1.01	0.98–1.05	0.97	0.89–1.07	0.96	0.87–1.06
CTQ	1.00	0.97–1.04	1.00	0.97–1.04	1.05	0.98–1.11	1.00	0.98–1.03	1.05	0.99–1.11	1.04	0.98–1.11
BHS	1.18***	1.08–1.29	1.13*	1.01–1.26	0.91	0.75–1.11	0.95	0.86–1.05	0.77**	0.63–0.94	0.81*	0.66–0.99
CDRS-S	1.02	0.96–1.09	1.01	0.93–1.09	0.94	0.80–1.09	0.99	0.93–1.04	0.92	0.79–1.07	0.93	0.79–1.09
NSSI	1.29**	1.08–1.53	1.45***	1.20–1.75	1.25	0.91–1.74	1.13*	1.00–1.27	0.98	0.72–1.33	0.87	0.63–1.18
Substance use	1.15	0.88–1.51	1.29	0.95–1.75	1.17	0.74–1.85	1.12	0.90–1.40	1.01	0.64–1.59	0.90	0.56–1.45
Gender	2.63	0.94–7.36	1.41	0.38–5.19	0.62	0.08–4.80	0.54	0.19–1.55	0.24	0.30–1.89	0.44	0.05–4.04

BAI, Beck anxiety inventory; CTQ, childhood trauma questionnaire; BHS, Beck hopelessness scale; CDRS-S, children's depression rating scale minus suicide items; NSSI, non-suicidal self-harm; NO, non-suicidal group; ID, suicidal ideator group; AT+, ideator-attempter group; AT−, lifetime attempter group.

* *p* < .05.

** *p* < .01.

*** *p* < .001.

Mediation and moderation analyses were performed using the Process makro v. 3.5 from Hayes (70). For the serial mediation analysis (model 6), the standardized childhood trauma scores were entered as the antecedent for suicidal ideation, and hopelessness and depression severity as the two mediators. Process uses a standard bootstrapping approach that provides confidence intervals for indirect effects. 10,000 bootstrapping samples were used to estimate the indirect effect, together with HC3 (Davidson-MacKinnon) heteroscedasticity-consistent interference parameters (71). Confidence intervals that do not include 0 provide evidence for a statistically significant effect. Gender was added as a covariate. IBM SPSS version 27 was used to perform all statistical analyses, with *p*-values set at 0.05 significance level.

## Results

### Sociodemographic and descriptive results

Supplementary Table 4 in the supplementary information summarizes the sociodemographic and descriptive statistics of the patients based on the presence of suicidal ideation and attempts. Ideator-attempters (AT+: *N* = 48, 85.7%) and lifetime attempters (AT−: *N* = 10, 83.3%) were more likely to be female compared to ideators (ID: *N* = 73, 71.6%) and the non-suicidal group (NO: *N* = 49, 64.5%; *χ^2^* = 8.18, *p* = .042). Furthermore, 80.4% (*N* = 45) of the ideator-attempters (AT+) and 75% (*N* = 9) of the lifetime attempters without current suicidal thoughts (AT−) had previously been hospitalized compared to 49% (*N* = 50) and 52.6% (*N* = 40) of the ideators and non-suicidal youths, respectively (*χ*^2^ = 17.22, *p* < .001). Otherwise, the groups did not differ in terms of demographic characteristics or illness history variables such as age of onset, illness duration, antidepressant medication or recurrent version of the illness.

### Clinical characteristics

Suicidal ideators (ID; *M* = 52.55, *SD* = 7.69) and ideator-attempters (AT+; *M* = 52.59, *SD* = 7.31) had higher depression severity (CDRS-S = total score corrected for suicide items) compared to the non-suicidal group [NO; *M* = 48.51, *SD* = 6.33; *F* (3,241) = 6.00, *p* < .001, *n*^2^ = .069], controlled for gender (see Table 2; Supplementary Table 4). There was a significant main effect of gender [*F*(1) = 10.10, *p* = .002, *n^2^* = .04], with females (*M* = 52.13, *SD* = 7.44) exhibiting significantly higher depression scores than males (*M* = 48.58, *SD* = 7.90). Furthermore, ideators (ID; *M* = 14.38, *SD* = 4.38) and ideator-attempters (AT+: *M* = 14.29, *SD* = 4.33) were more hopeless than non-suicidal youths (NO: *M* = 8.82, *SD* = 4.64) and lifetime attempters without current suicidal ideation (AT−: *M* = 7.33, *SD* = 5.07), after adjustment for depression severity (CDRS-S) and gender [*F*(3,227) = 23.93, *p* < .001, *n^2^* = 0.24]. Similarly, patients with suicidal ideations (ID: *M* = 23.17, *SD* = 11.49) and the ideator-attempters (AT+: *M* = 26.80, *SD* = 13.20) were more anxious compared to non-suicidal ones (NO: *M* = 16.39, *SD* = 11.57), adjusted for depression severity (CDRS-S) and gender [*F*(3,239) = 6.07, *p* < .001, *n^2^* = 0.07]. In contrast, the non-suicidal youths showed higher resilience scores (NO: *M* = 46.28, *SD* = 14.78) than ideators (ID: *M* = 34.07, *SD* = 11.59) and ideator-attempters (AT+: *M* = 36.57, *SD* = 12.95), also controlled for depression severity (CDRS-S) and gender [*F*(3,237) = 9.98, *p* < .001, *n^2^* = 0.11]. Attempters with and without current suicidal ideation differed in their hopelessness and anxiety scores (see Supplementary Table 4).

**Table 2:** Clinical symptom pattern (mean and SD) of the sample based on the presence of suicidal thought and behavior, controlled for gender.

Depression symptoms (CDRS-R)	NO	ID	AT+	AT-	Male	Female	MANOVA/ANOVA		*Post-hoc* comparison	
							Group	Gender	Group	Gender
School work	3.95 (1.27)	3.94 (1.59)	3.98 (1.51)	3.67 (1.16)	3.72 (1.62)	4.07 (1.38)				
Anhedonia	3.89 (1.21)	4.18 (1.20)	4.23 (1.32)	4.08 (0.79)	4.38 (1.42)	4.02 (1.15)		*p* = .018, *n*^2^ = .023		**ns**
Social withdrawl	3.33 (1.54)	3.49 (1.38)	3.21 (1.63)	3.08 (1.08)	3.51 (1.68)	3.32 (1.43)				
Sleep problems	3.58 (1.43)	4.13 (1.08)	3.84 (1.35)	4.08 (1.08)	3.61 (1.32)	4.00 (1.23)	*p* = .045, *n*^2^ = .033	*p* = .032, *n*^2^ = .019	**NO<ID**	**M<F**
Appetite	2.61 (1.30)	2.97 (1.21)	2.95 (1.34)	2.83 (1.19)	2.67 (1.30)	2.89 (1.26)				
Tiredness	4.88 (1.24)	5.19 (1.18)	5.13 (1.19)	4.67 (1.44)	4.48 (1.55)	5.20 (1.10)		*p* < .001, *n*^2^ = .058		**M<F**
Physical complaints	2.75 (1.44)	2.72 (1.33)	2.75 (1.39)	2.92 (1.73)	2.45 (1.30)	2.86 (1.42)		*p* = .035, *n*^2^ = .018		**M<F**
Irritability	4.05 (1.34)	3.58 (1.39)	3.41 (1.41)	3.42 (1.56)	3.72 (1.54)	3.65 (1.37)	*p* = .036, *n*^2^ = .035		**ns**	
Guilt	2.55 (1.48)	2.99 (1.47)	3.48 (1.43)	3.17 (1.19)	2.48 (1.39)	3.18 (1.50)	*p* = .020, *n*^2^ = .040	*p* = .006, *n*^2^ = .031	**NO<AT+**	**M<F**
Self-esteem	3.86 (1.46)	4.86 (1.40)	5.13 (1.35)	3.42 (1.51)	4.39 (1.36)	4.61 (1.58)	*p* < .001, *n*^2^ = .139		**NO/AT-<ID/AT+**	
Depressed mood	4.43 (0.90)	4.95 (0.91)	4.93 (0.89)	4.33 (0.99)	4.67 (1.05)	4.80 (0.90)	*p* < .001, *n*^2^ = .070		**NO<ID/AT+**	
Crying	3.39 (1.90)	4.25 (1.74)	4.18 (1.74)	3.25 (2.05)	2.81 (1.57)	4.34 (1.78)	*p* = .009, *n*^2^ = .046	*p* < .001, *n*^2^ = .123	**NO<ID**	**M<F**
Facial affect	2.11 (0.92)	2.16 (0.94)	2.27 (1.09)	1.92 (1.00)	2.23 (1.11)	2.18 (0.96)				
Listless speech	1.57 (0.70)	1.48 (0.75)	1.52 (0.76)	1.92 (1.00)	1.70 (0.85)	1.47 (0.70)		*p* = .043, *n*^2^ = .017		**F<M**
Hypoactivity	1.57 (0.77)	1.67 (0.99)	1.59 (0.97)	1.33 (0.65)	1.77 (1.11)	1.56 (0.87)				
Total score										
depression severity (CDRS-S)	48.51 (6.33)	48.51 (6.33)	52.59 (7.31)	47.50 (5.52)	48.58 (7.90)	52.13 (7.44)	*p* < .001, *n*^2^ = .074	*p* = .002, *n*^2^ = .040	**NO<ID/AT+**	**M<F**

CDRS-R, children's depression rating scale—revised; CDRS-S, children's depression rating scale minus the two items related to suicidal/morbid ideation; NO, non-suicidal group; ID, ideators; AT+, ideator-attempter; AT-, lifetime attempters; ns, non-significant; M, male; F, female. Bold font indicates significant *post-hoc* comparison (*p* < .05).

In addition, ideator-attempters (AT+: *M* = 44.96, *SD* = 13.81) scored higher on the *Childhood Trauma Questionnaire* than non-suicidal youths [NO: *M* = 37.58, *SD* = 9.58, *F*(3,201) = 2.89, *p* = .036, *n^2^* =. 091], and there was a trend toward a higher number of abuse subtypes in the ideator-attempter group [*H*(3) = 6.40, *p* = 0.09].

Regarding self-injurious behavior, young patients with a history of suicide attempts and suicidal ideation (AT+) had the highest frequency of self-harm behavior (*M* = 5.89, *SD* = 3.75), followed by ideators (ID: *M* = 3.98, *SD* = 3.30), lifetime attempters (AT−: *M* = 2.00, *SD* = 2.63) and then the non-suicidal youths [NO: *M* = 1.41, *SD* = 2.23; *H* (3) = 56.65, *p* < .001]. They were also more likely to use a variety of methods to hurt themselves [*H*(3) = 52.91, *p* < .001], and to use potentially more harmful methods such as burns and deep cuts (see Supplementary Table 4). While lifetime attempters without current suicidal ideation (AT−) did not have a high frequency of self-harm behavior in the past month, 75% of them had committed self-harm in the past year.

Attempters also indicated having experimented with a higher number of different drugs (lifetime substance abuse score: AT+: *M* = 2.77, *SD* = 1.90; AT−: *M* = 2.58, *SD* = 1.98) compared to ideators (ID: *M* = 2.11, *SD* = 1.65) and non-suicidal youths [NO: *M* = 1.49, *SD* = 1.68; *H*(3) = 18.12, *p* < .001]. They also scored higher on the global substance abuse risk score [*F* (3, 45.78) = 5.03, *p* = .004, *n^2^* ^=^ .063], and were most likely to have experimented with smoking, alcohol and cannabis.

### Clinical symptom pattern

**Figure 1:**
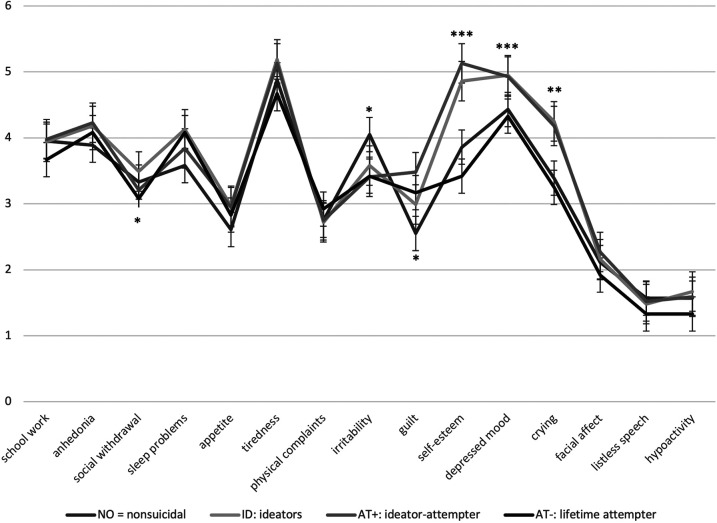
Depression symptom patterns according to the four groups. *p < .05; **p < .01; ***p < .001.

In a next analysis, the clinical symptom profile was compared between the four groups by running a multivariate ANOVA adjusted for gender (see Figure 1; Table 2). Overall, the four groups differed significantly in their symptom pattern [*F*(45, 675.14) = 1.97, *p* < .001, *n^2^* = 0.12], namely in the symptoms sleeping problems, irritability, guilt, self-esteem, depressed mood and crying. Compared to the non-suicidal group, ideators had more problems sleeping (*M* = 4.13, *SD* = 1.08), had lower self-esteem (*M* = 4.86, *SD* = 1.40), a more severely depressed mood (*M* = 4.95, *SD* = 0.91), and cried more often (*M* = 4.25, *SD* = 1.74). Ideator-attempters had more guilt (*M* = 3.48, *SD* = 1.43), a lower self-esteem (*M* = 5.13, *SD* = 1.35) and a more severely depressed mood (*M* = 4.93, *SD* = 0.89) compared to the non-suicidal group (guilt: *M* = 2.55, *SD* = 1.48; self-esteem: *M* = 3.86, *SD* = 1.46; depressed mood: *M* = 4.43, *SD* = 0.90). Suicidal ideators (*M* = 4.86, *SD* = 1.40) and ideator-attempters (*M* = 5.13, *SD* = 1.35) also had a lower self-esteem compared to the lifetime attempters without current suicidal ideation (AT−; *M* = 3.42, *SD* = 1.51). However, there was no depression symptom which differentiated between suicidal ideators (ID) and ideator-attempters (AT+) or between the non-suicidal group (NO) and the lifetime attempters without current suicidal ideation (AT−). There was a significant main effect of gender [*F*(15, 228) = 4.77, *p* < .001, *n^2^* = .24]. Females showed significantly higher sleeping problems (*M* = 4.00, *SD* = 1.23), tiredness (*M* = 5.20, *SD* = 1.10), physical complaints (*M* = 2.86, *SD* = 1.42), guilt (*M* = 3.18, *SD* = 1.50) and cried more often (*M* = 4.34, *SD* = 1.78) compared to males (sleeping problems: *M* = 3.61, *SD* = 1.32; tiredness: *M* = 4.48, *SD* = 1.55; physical complaints: *M* = 2.45, *SD* = 1.30; guilt: *M* = 2.48, *SD* = 1.39; crying: *M* = 2.81, *SD* = 1.57), whereas males showed higher values in listless speech (*M* = 1.70, *SD* = 0.85) compared to females (*M* = 1.47, *SD* = 0.70; see Table 2).

### Multivariate analyses

In the next analysis, a multivariate logistic omnibus analysis was conducted to determine which risk factors differentiated most between the four groups, entering gender, hopelessness, BAI total score, CTQ total score, depression severity, drug use and self-harm frequency into the model. The overall model significantly predicted group membership [*χ^2^*(21) = 80.82, *p* < .001, Nagelkerke *R^2^* = 0.39]. The unique effects of all predictors are shown in Table 1. Of all these predictors, only hopelessness [*χ*^2^(3) = 19.19, *p* < .001] and self-harm [*χ*^2^(3) = 17.97, *p* < .001] proved to be significant. Using the non-suicidal adolescents as the reference group, ideators had an OR of 1.18, 95% CI [1.08, 1.29] for increased hopelessness and an OR of 1.29, 95% CI [1.08, 1.53] for self-harm. Suicide ideator-attempters had an OR of 1.13, 95% CI [1.01, 1.26] for hopelessness and an OR of 1.45, 95% CI [1.20, 1.75] to engage in self-harm behavior compared to the non-suicidal group. Compared to the ideators, ideator-attempters had an OR of 1.13, 95% CI [1.00, 1.27] for self-harm. There was no variable distinguishing between the non-suicidal group and the lifetime attempter group without current suicidal ideation. Compared to the lifetime attempters with current suicidal ideation, those without suicidal thoughts had an OR of 0.81, 95% CI [0.66, 0.99] for hopelessness.

The results of the two additional multivariate regression models are presented in the supplementary information (see Supplementary Table 2 for the full model including all variables, and Supplementary Table 3 for the model where resilience was included but hopelessness was excluded). Both models reached overall significance in predicting group membership [full model: *χ²*(24) = 83.74, *p* < .001, Nagelkerke *R*² = 0.42; model including resilience but excluding hopelessness: *χ²*(21) = 73.83, *p* < .001, Nagelkerke *R*² = 0.38]. The unique effects of the predictors changed only slightly (see Supplementary Tables 2, 3), with the only significant changes observed in self-harm not distinguishing significantly anymore between ideators and ideator-attempters (ID vs. AT+) in the full model and gender yielding a significant effect in both models when comparing those with no current suicidal ideation to those with suicidal ideation (NO vs. ID). However, it is important to note that the overall direction of the effects remained unchanged.

### Hopelessness as a mediator between childhood maltreatment and suicidal ideation

**Figure 2:**
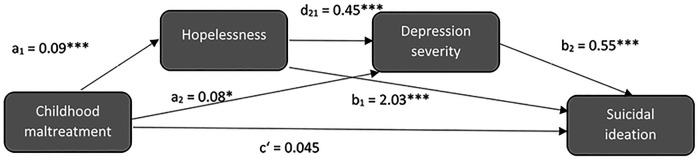
Serial mediation analysis.

A serial mediation model was performed to assess whether hopelessness and depression severity mediate the relationship between childhood maltreatment and suicidal ideation (see Figure 2; Supplementary Table 5). Childhood trauma did not have a direct effect on suicidal ideation [*β* (c') = 0.045; CI: −0.153; ULCI: 0.243]. However, CTQ scores were associated with BHS scores (a_1_ = 0.095, *p* < .001) and with CDRS-S scores (a_2_ = 0.079, *p* = 0.036). BHS scores in turn were associated with CDRS-S scores (d_21_ = 0.449, *p* < .001) and SIQ scores (b_1_ = 2.033, *p* < .001). In addition, CDRS-S scores also predicted SIQ scores (b_2_ = 0.549, *p* < .001). Bootstrapping analyses over the whole pathways indicated that hopelessness significantly mediated the direct path between childhood maltreatment and suicidal ideation [*β* (a_1_b_1_) = 0.117, SE = 0.033, LLCI: 0.054; ULCI: 0.184] as well as the serial path between childhood maltreatment, depression severity and suicidal ideation [*β* (a_1_d_21_b_2_) = 0.014, SE = 0.007, LLCI: 0.004; ULCI: 0.029]. Furthermore, the serial path between childhood maltreatment, depression severity and suicidal ideation was also significant [*β* (a_2_b_2_) = 0.026, SE = 0.015, LLCI: 0.002; ULCI: 0.059], indicating that childhood maltreatment also increases suicidal ideation by increasing depression severity, independent of hopelessness.

### Self-harm as a moderator between suicidal ideation and suicide attempts

**Figure 3:**
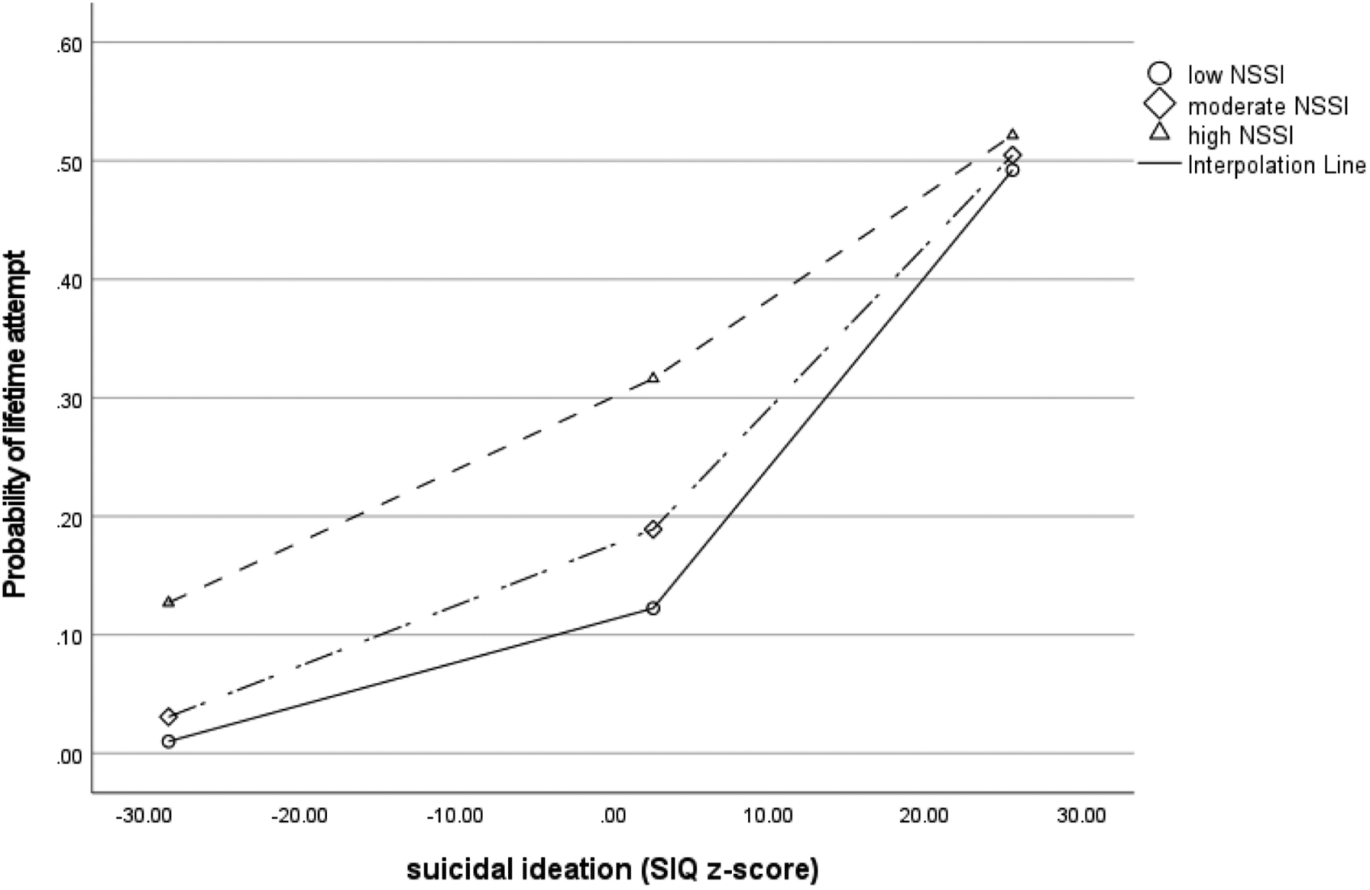
Moderation analysis: self-harm moderates the association between suicidal ideation and probability of having made a suicide attempt.

Self-harm was the main variable that distinguished between suicidal ideators and ideator-attempters in the multivariate analysis. To explore this relationship further, it was hypothesized that self-harm might moderate the relationship between suicidal ideation and attempts. For this analysis, the group with lifetime history of suicide attempt but no current suicidal ideation was not considered. Logistic regression analysis was significant *(χ*^2^ = 56.17, *p* < .001, Nagelkerne = 0.34) with suicidal ideation (*β* = 0.06, SE = 0.01, *p* < .001), self-harm (*β* = 0.19, SE = 0.08, *p* = 0.01) and the interaction between suicidal ideation and self-harm (*β* = −0.01, SE = 0.01, *p* = 0.03) predicting the likelihood of a suicide attempt. As shown graphically in Figure 3, self-harm increased the probability of a suicide attempt in patients with low or moderate suicidal ideations. However, for adolescents with very frequent suicidal ideations, the probability of a suicide attempt was high, regardless of self-harm behavior.

## Discussion

In the sample of this study, about two-thirds of pediatric MDD patients reported frequent suicidal ideations and about a quarter reported having attempted suicide in their lifetime, illustrating the high risk of suicidal behavior in this patient group. In a Canadian clinical adolescent sample, only 11% of depressed adolescents did not exhibit suicidal thoughts or behaviors (72) although lower percentages have been reported in other cultures (5). Suicidality in adult patients is associated with the onset of the depressive illness in adolescence (73) and 20% of depressed adolescents will attempt suicide by the age of 30 (6). A large population based cohort-study in Sweden including 1.5 million adults showed that those who suffered from depression in adolescence had a 14 times higher risk of dying by suicide than those who did not suffer from depression (74). Therefore, understanding the factors related to suicidality in depressed children and adolescents is important for suicide prevention and improving clinical care. Using a large sample, this study investigated whether a wide range of demographic and clinical parameters differentiated between groups of non-suicidal depressed youths, suicidal ideators and suicide attempters. In this study, suicide attempters were disproportionately female and more likely to having had previous inpatient treatment, with no other sociodemographic variables differentiating between groups.

### Clinical characteristics and symptom pattern

Patients with frequent suicidal ideation in the past month had higher depression severity than patients not currently experiencing suicidal thoughts, regardless of previous suicidal behavior. Chesin and Cascardi (75) reported similar findings when assessing lifetime suicide attempts and suicidal ideation in a large group of 780 undergraduate students. Depression severity was highest in the group with a history of suicide attempts and current suicidal ideation, and lower in those who had attempted suicide previously compared to young adults with current suicidal ideation.

When comparing the clinical symptom pattern of the four groups, it became evident that the difference in depression severity was not due to an overall higher severity in all but rather to a higher severity in specific depression symptoms. For example, suicidal ideation, regardless of a previous history of suicide attempts, was associated with a more severely depressed mood and lower self-esteem. In adolescence, the development of a stable self is one of the main developmental tasks, and failure to build self-esteem is associated with the emergence of suicidal ideations. In a longitudinal study of 240 youths aged 8–16 years, implicit self-esteem predicted emergence of suicidal ideations several years later (76). In addition, a large scale Korean study including 2,964 persons of varying ages indicated that self-esteem was associated with suicidal ideation regardless of age or depressive symptoms (77).

In the sample of this study, having suicidal thoughts was also linked to sleeping problems and frequent crying. The association between sleeping problems, especially insomnia, and suicidal thoughts and behaviors has been shown in adolescent and adult populations (78, 79), although it is less clear whether insomnia precedes suicidal thoughts or is a consequence of them. It is possible that sleep dysregulation affects executive control (80), increasing the risk for suicidal behavior.

Patients with suicidal thoughts and a history of suicide attempts showed more shame and guilt compared to the non-suicidal group. In a systematic review, shame and guilt have both been associated with self-harm and suicidal behavior, although the authors conclude that more research is needed (81). In a qualitative study, asking adolescents why they had attempted suicide, guilt and shame were among the intrapersonal factors identified as contributing to suicide attempts (82).

Even more, the patients with current frequent suicidal ideation also showed increased hopelessness, anxiety, substance abuse and self-harm behavior together with reduced resilience compared to non-suicidal youths, even when controlling for depression severity. Generally, depressed adolescents with frequent suicidal thoughts, regardless of a lifetime history of suicide attempts, appear to be more severely ill and more likely to exhibit symptoms other than the core depressive symptoms than those without suicidal ideation. This co-occurrence of various symptoms of different psychopathology might be especially detrimental for suicidal behavior. In a study on 1,287 high school drop-outs, depression and hopelessness were directly associated with suicidal behavior, but anxiety increased the levels of depression and hopelessness (83). Similarly, in a large study on 507 adolescents who had a lifetime history of suicide attempts, both depression and anxiety were associated with suicidal ideation through their detrimental effects on social functioning (84). That is, the interplay of different psychopathological symptoms might exacerbate the effects on daily functioning to such a degree that the emergence of suicidal thoughts and behaviors becomes more likely.

The findings that non-suicidal adolescents show higher resilience than those with suicidal thoughts or behavior is in line with current literature. For instance, Xu et al. (85) found that adolescents with suicidal ideation (*n* = 49) had lower resilience scores compared to those without suicidal ideation (*n* = 50). A systematic review by Shahram et al. (86) emphasizes the importance of internal (e.g., coping skills) and external resilience (e.g., social support) factors for suicide prevention, though more research is needed to understand the mechanisms involved. Several studies found resilience to act as a mediator. Chang et al. (87) found resilience to mediate the relationship between sleep problems and suicidal ideation in female adolescents. In a sample of 3,146 Chinese adolescents, Chen et al. (28, 29) demonstrated that resilience, particularly emotion regulation and social support, mediated the effect of childhood maltreatment on suicidal ideation. Additionally, resilience has been shown to moderate the effect of academic stress on suicidal ideation (88).

In the sample of this study, lifetime attempters without current suicidal ideation did not show any differences regarding clinical characteristics compared to the non-suicidal group. While this result needs to be interpreted cautiously given the small number in the group of lifetime attempters without current suicidal ideation, it highlights the intra-individual variability of suicidal ideation. In some patients, suicidal ideation is elevated but declines rapidly, but in a subgroup suicidal thoughts are chronically elevated and only decline very slowly (89, 90). In adults, suicidal thoughts and depressive symptoms seem to follow unique trajectories. That is, 16% of 400 adult patients with depression showed a remission in depression symptoms but not in suicidal ideation over a 12 months follow-up period. On the other hand, 18% of the same sample had decreasing suicidal ideation but no change in depression symptoms during the follow-up period, suggesting that although suicidal thinking is related to depression symptoms, it is also influenced by other factors (91).

### The role of hopelessness

In the conducted multivariate logistic analysis, hopelessness was the variable that was most closely associated with suicidal thoughts. Previous studies also reported hopelessness to be one of the main risk factors for suicidal ideation (33, 47), and for dying by suicide (36). According to the hopelessness theory of depression and suicide (30, 31), hopelessness is one of the mechanism which leads to depression and suicidal thoughts, especially in cases of childhood maltreatment. Similar to adult samples (25), about half of the patients in the sample of this study reported at least one form of childhood maltreatment, and suicide attempters with current suicidal ideation reported overall higher childhood trauma scores than non-suicidal youths.

The serial mediation analysis confirmed the hypothesis of the hopelessness theory of depression, given that hopelessness mediated the relationship between childhood maltreatment and suicidal ideation, not only directly but also indirectly over depression severity. Similarly, in a study on 297 undergraduates, hopelessness partially mediated the relationship between emotional abuse and suicidal ideation over a 2.5 years follow-up period (92). In a study on 1,287 high-school drop-outs, depression and hopelessness mediated the relationship between school performance, family support and suicidal risk behavior (83). Further, hopelessness has been identified as mediator between impulsivity (93), self-esteem and social anxiety (94) and work stress (95) and suicidal ideation in (young) adult populations.

In the sample of this study, childhood maltreatment scores were also directly associated with depression severity, suggesting that childhood maltreatment might not only affect depression severity through its effect on cognitive schemata such as hopelessness, but also by affecting other core depression symptoms, such as for example self-esteem. In a large study on out-of-home placed adolescents, for example, self-esteem mediated the relationship between emotional abuse and depressive symptoms (96). And in a review, high self-esteem was found to be a resilience factor protecting against adverse effects of childhood maltreatment on psychological wellbeing in young people (97).

### Differences between ideators and attempters: the role of non-suicidal self-harm behavior

In the sample of this study, lifetime suicide attempters with frequent suicidal ideation engaged in self-harm behavior more often compared to those with suicidal thoughts only. In adolescence, self-harm behavior has become more frequent in recent years (98, 99) with lifetime prevalence estimated to lie between 17% and 22% in the general population (1, 100). In clinical samples, about two thirds of minors engage in self-harm behavior (101), with even higher rates in those seeking help for suicidal behavior (102). While non-suicidal self-harm is by definition done without the intent to die, there is a close link to suicidal behavior. Studies using diaries to assess suicidal ideation and NSSI on a daily basis show that adolescents are more likely to perform NSSI on days when they are having suicidal thoughts, with the adolescents giving coping with suicidal thoughts as one of the main explanations why they engaged in NSSI in the first place (103). Furthermore, adolescents who performed NSSI on a given day were 2.5 times more likely to attempt suicide compared to adolescents who did not perform NSSI on that day (104). In over 6,000 college students NSSI was associated with the transitioning from suicidal ideations to plans, as well as to attempts among those who already have a suicide plan (105). Importantly, cessation of NSSI also reduces subsequent suicidal behavior (106), making it an important focus for suicide prevention and therapy. The results of this study corroborate these findings, showing an OR of 1.13 for self-harm in ideator-attempters compared to ideators only and an OR of 1.45 compared to non-suicidal youths. Furthermore, self-harm behavior moderated the relationship between suicidal ideation and attempts, so that a young person with low to moderate suicidal ideations was more likely to having attempted suicide previously when also engaging in frequent self-harm behavior. When suicidal ideations were very high, the frequency of self-harm did not have any impact on the probability for a suicide attempt. In these cases, the risk of a suicide attempt was high irrespective of the self-harm behavior.

According to the IPTS (42), suicide attempts occur when the desire to die is combined with the capability to act on it. The capability is acquired by experiences of pain. In line with this theory, the suicide attempters in the sample of this study not only engaged in self-harm behavior more frequently but also used potentially more hurtful methods such as burning and deep cutting. Indeed, not only severity but also diversity of self-harm behavior was associated with suicide attempts in a large sample of 628 Portuguese adolescents and young adults (107). Interestingly, the only difference between the non-suicidal youths and the small group of lifetime suicide attempters but no current suicidal ideation observed in the current study was that the youths with a history of suicide attempts had a higher lifetime substance abuse score compared to the non-suicidal group, indicating that they had experimented with a higher variety of drugs. As drug use can also be seen as some sort of self-harm, this also points to the important relationship between harming one-self and attempting suicide.

### Limitations

The main limitation of this study is its cross-sectional design, which does not allow assessing which behavior or symptom emerged antecedent to the others. It would be interesting to assess variations in hopelessness, depression severity, and non-suicidal and suicidal self-harm behavior over an extended period of time, to get a better understanding of how these variables interact with each other over time. In addition, through recording of the exact date and numbers of previous suicide attempts, it would have been possible to establish whether time between suicide attempt and the time of assessment had any influence on the relationships of the constructs assessed in this study. Furthermore, although a large sample of 246 depressed children and adolescents was included, the sample was somewhat biased because of the inclusion criteria of the omega-3 fatty acid trial. For example, only moderately to severely depressed patients were included, and because of language barriers, patients of migrant families might be underrepresented in the current sample. Moreover, this study did not allow for a deeper examination of the interplay between hopelessness and resilience. Future research should explore this relationship in larger sample sizes. Finally, although the effects of antidepressant use among suicidality groups were not statistically significant, the possibility that they indirectly influence suicidality and other variables of interest cannot be definitively ruled out. Previous studies have highlighted a potential link between antidepressant medication and suicidality, particularly in relation to suicidal behavior [e.g., the “black box warning,” as discussed in the meta-analysis by (108)]. Further research is needed to explore the role of antidepressants in relation to suicidal behaviors, as this could provide valuable insights into potential indirect effects or interactions.

## Conclusion

This study shows that suicidal behavior in youths is associated with a distinctive clinical symptom pattern characterized by increased sleeping problems, more guilt, low self-esteem, higher depressed mood and more crying. Furthermore, suicidal adolescents also experienced other than the core depression symptoms, such as anxiety and low resilience. Hopelessness was the factor that was mostly associated with suicidal ideation while self-harm behavior distinguished between those who think about suicide and those who attempt it. Given the rising prevalence of self-harm behavior in youths, prevention and treatment strategies for self-harm behavior in youths should be further explored as an effective prevention strategy for suicide in young people.

## Data Availability

The raw data supporting the conclusions of this article will be made available by the authors, without undue reservation.

## The Omega-3 Study Team

The Omega-3 Study Team contributed with implementation of the design with following roles: Sponsor-investigator of the trial is GB (Department of Child and Adolescent Psychiatry, University Hospital of Psychiatry, University of Zurich, Neumünsterallee 9, 8,032 Zurich, Switzerland; gregor.berger@pukzh.ch; +41-43-384-66-66). Chief investigator is KS. IH is study coordinator. Principal investigators and research psychologists from the clinical sites are as follows: Research psychologists: Noemi Baumgartner, Sophie Emery, Mona Albermann, and Kristin Nalani (Department of Child and Adolescent Psychiatry, University Hospital of Zurich); Principal Investigator Basel: KS; Investigators and research psychologists: Oliver Pick, Alain Di Gallo, and Michael Strumberger (Department of Child and Adolescent Psychiatry, Psychiatric University Hospitals Basel); Principal Investigator Basel-Stadt: Brigitte Contin; Investigator: Stefan Müller (Child and Adolescent Psychiatric Services Baselland); Principal Investigator: Silke Bachmann and Lars Wöckel, Investigator: Simone Heitzer (Clienia Littenheid); Principal Investigator: Bruno Rhiner; Investigators: Amir Yamini (Child and Adolescent Psychiatric Services Thurgau); Principal Investigator: Suzanne Erb; Investigators: Michael Schmid (Child and Adolescent Psychiatric Services St. Gallen); Principal Investigator: Ulrich Müller-Knapp; Investigator: Ioannis Christodoulakis (Klinik Sonnenhof). Ulrike Held and Burkhardt Seifert (retired) are statistical consultants. Edna Grünblatt is head of the department for translational molecular psychiatry (Department of Child and Adolescent Psychiatry, University Hospital of Zurich). Martin Hersberger is head of the division of Clinical Chemistry and Biochemistry at the University Children's Hospital Zürich and his PhD student Ivan Hartling of the division of Clinical Chemistry and Biochemistry who will analyze the bioactive lipids; Romuald Brunner (University of Heidelberg), Jürgen Drewe (University of Basel), and Julia Braun (Epidemiology, Biostatistics, and Prevention Institute, University of Zürich) are members of the Data Monitoring Committee. Jenny Peterson, Clinical Trials Pharmacy (Kantonsapotheke) Zürich, responsible for the packaging, handling, and quality of the study medication.
