# Supplementary material for: Clinical characteristics of depressed children and adolescents with and without suicidal thoughts and behavior: a cross-sectional study
Source: Front Child Adolesc Psychiatry. 2025 Feb 21;4:1510961. doi: 10.3389/frcha.2025.1510961 (PMC11885278; doi:10.3389/frcha.2025.1510961)
Supplement: Supplementary file 1 [file Datasheet1.pdf]

SI Table 1: Correlation matrix of the predictors in the multinomial logistic regression analyses.

	Gender	Depression severity (CDRS-S)	Hopelessness	Resilience	Anxiety	CTQ total score	Self-harm frequency	Substance use
**Gender**	r = 1							
**Depression severity (CDRS-S)**	r = .205**	r = 1						
**Hopelessness**	r = .119	r = .334***	r = 1					
**Resilience**	r = -.233***	r = -.293***	r = -.695***	r = 1				
**Anxiety**	r = .370***	r = .352***	r = .331***	r = -.296***	r = 1			
**CTQ total score**	r = .107	r = .206**	r = .258***	r = -.212**	r = -.292***	r = 1		
**Self-harm frequency**	r = .237***	r = .251***	r = .387***	r = -.282***	r =.286***	r = .119	r = 1	
**Substance use**	r = .059	r = -.039	r = .102	r = -.103	r =.107	r =.214**	r =.199**	r = 1

Note: r = Pearson Correlation. CDRS-S = Children's Depression Rating Scale minus suicide items; CTQ = Childhood Trauma Questionnaire; * p < .05; ** p < .01; *** p < .001.

SI Table 2: Multinomial logistic regression of variables associated with the groups based on suicidal ideation and behavior; full model including hopelessness and resilience.

	**NO vs ID**		**NO vs AT+**		**NO vs AT-**		**ID vs AT+**		**ID vs AT-**		**AT+ vs AT-**	
	OR	95% CI	OR	95% CI	OR	95% CI	OR	95% CI	OR	95% CI	OR	95% CI
BAI	1.01	0.97 – 1.06	1.03	0.98 –1.08	1.01	0.92 – 1.12	1.02	0.98 – 1.05	1.00	0.91 – 1.10	0.99	0.89 – 1.09
CTQ	1.00	0.96 – 1.04	1.01	0.97 – 1.05	0.98	0.88 – 1.08	1.01	0.98 – 1.04	0.98	0.89 - 1.08	0.97	0.87 – 1.07
BHS	1.14*	1.01- 1.28	1.16*	1.00 – 1.34	0.84	0.65 – 1.09	1.02	0.90 – 1.15	0.74*	0.57 - 0.96	0.73*	.55 – 0.96
CD-RISC	1.02	0.93 – 1.01	1.01	0.96 – 1.06	0.97	0.90 – 1.06	1.04	1.00 – 1.28	1.00	0.92 – 1.09	0.97	0.89 – 1.06
CDRS-S	1.27	0.95 – 1.09	1.03	0.93 – 1.09	0.92	0.77 – 1.10	0.99	0.93 – 1.05	0.91	0.76 – 1.08	0.92	0.76 – 1.10
NSSI	1.17*	1.06 – 1.52	1.01***	1.18 – 1.75	1.24	0.88 – 1.77	1.13	1.00 – 1.28	0.98	0.67 – 1.37	0.87	0.61 – 1.22
Substance use	1.17	0.89 – 1.55	1.28	0.94 – 1.75	1.16	0.72 – 1.87	1.09	0.86 – 1.38	0.99	0.62 – 1.60	0.91	0.56 – 1.50
Gender	2.94*	1.01 – 8.55	1.42	0.37 – 5.37	0.95	0.11 – 8.22	0.48	0.16 – 1.42	0.32	0.04 – 2.94	0.67	0.06 – 6.95

Note: * p < .05; ** p < .01; *** p < .001; BAI = Beck Anxiety Inventory; CTQ = Childhood Trauma Questionnaire; BHS = Beck Hopelessness Scale; CD-RISC = Connor-Davidson-Resilience Scale; CDRS-S = Children’s Depression Rating Scale minus suicide items; NSSI = non-suicidal self-harm; NO = non-suicidal group; ID = suicidal ideator group; AT+ = ideator-attempter group; AT- = lifetime attempter group.

SI Table 3: Multinomial logistic regression of variables associated with the groups based on suicidal ideation and behavior; model including resilience but excluding hopelessness.

	NO vs ID		NO vs AT+		NO vs AT-		ID vs AT+		ID vs AT-		AT+ vs AT-	
	OR	95% CI	OR	95% CI	OR	95% CI	OR	95% CI	OR	95% CI	OR	95% CI
BAI	1.02	0.98 – 1.06	1.03	0.98 – 1.08	0.10	0.91 – 1.09	1.02	0.98 – 1.05	0.98	0.90 – 1.07	0.97	0.88 – 1.06
CTQ	1.00	0.97 – 1.04	1.02	0.97 – 1.06	0.97	0.89 – 1.07	1.01	0.98 – 1.05	.97	0.89 – 1.07	0.96	0.88 – 1.05
CD-RISC	0.94***	0.91 – 0.98	0.98	0.94 – 1.01	1.01	0.94 – 1.08	1.03	1.00 – 1.07	1.07	1.00 – 1.14	1.03	0.96 – 1.11
CDRS-S	1.03	0.97 – 1.10	1.02	0.95 – 1.10	0.91	0.78 – 1.06	0.99	0.94 – 1.05	0.88	0.75 – 1.03	0.89	0.76 – 1.04
NSSI	1.34**	1.12 – 1.60	1.52***	1.24 – 1.84	1.17	0.83 – 1.64	1.14*	1.00 – 1.28	0.87	0.63 – 1.21	0.77	0.55 – 1.08
Substance use	1.20	0.91 – 1.56	1.30	0.96 – 1.76	1.13	0.69 – 1.84	1.09	0.86 – 1.37	0.94	0.58 – 1.53	0.87	0.53 – 1.43
Gender	3.34*	1.19 – 9.37	1.57	0.43 – 5.80	0.80	0.11 – 6.04	0.47	0.16 – 1.39	0.40	0.03 – 1.84	0.51	0.06 – 4.52

Note: * p < .05; ** p < .01; *** p < .001; BAI = Beck Anxiety Inventory; CTQ = Childhood Trauma Questionnaire; BHS = Beck Hopelessness Scale; CD-RISC = Connor-Davidson-Resilience Scale; CDRS-S = Children's Depression Rating Scale minus suicide items; NSSI = non-suicidal self-harm; NO = non-suicidal group; ID = suicidal ideator group; AT+ = ideator-attempter group; AT- = lifetime attempter group.

SI Table 4: Descriptive statistics of the sample based on the presence of suicidal thoughts and attempts.

	NO	ID	AT+	AT-	χ2 / F	Post-hoc comparison
	N(%) / M(SD)	N(%) / M(SD)	N(%) / M(SD)	N(%) / M(SD)		
N / %	76 (30.9)	102 (41.5)	56 (22.8)	12 (4.8)		
Gender (female)	49 (64.5%)	73 (71.6%)	48 (85.7%)	10 (83.3%)	**8.18***	NO < AT+
Age	15.47 (2.11)	15.96 (1.36)	15.65 (1.17)	15.47 (1.28)	1.51[b]	
Parental marital status						
never married	6 (7.9%)	2 (2.0%)	1 (1.8%)	1 (9.1%)	12.31	
married	34 (44.7%)	57 (55.9%)	36 (64.3%)	5 (45.5%)		
divorced /separated	29 (38.2%)	37 (36.3%)	18 (32.1%)	5 (45.5%)		
widowed	5 (6.6%)	4 (3.9%)	1 (1.8%)	0		
unknown	2 (2.6%)	2 (2.0%)	0	0		
Current treatment						
inpatient	24 (31.6%)	34 (33.3%)	30 (53.6%)	6 (50.0%)	9.29	
outpatient	48 (63.2%)	63 (61.8%)	24 (42.9%)	5 (41.7%)		
daytreatment	4 (5.3%)	5 (4.9%)	2 (3.6%)	1 (8.3%)		
Age of onset	13.78 (2.49)	14.19 (2.30)	13.50 (1.97)	12.97 (2.27)	1.88[b]	
Duration of illness	15.89 (19.78)	14.18 (13.45)	17.16 (14.30)	19.67 (14.83)	5.34[c]	
Recurrent (yes)	16 (23.9%)	26 (25.5%)	20 (35.7%)	5 (41.7%)	4.92	
Previous inpatient treatment	40 (52.6%)	50 (49.0%)	45 (80.4%)	9 (75.0%)	**17.22*** **	NO / ID < AT+
**Clinical characteristics**						
AD (antidepressant medication)	24 (31.6%)	41 (40.2%)	30 (53.6%)	6 (50.0%)	6.87	
**Comorbid disorders**						
ADHD	16 (21.1%)	14 (13.7%)	5 (8.9%)	0	6.20	

lifetime anxiety disorder	16 (21.1%)	29 (28.4%)	23 (41.1%)	3 (25.0%)	6.42	
Depression severity (CDRS-S)	48.51 (6.33)	52.55 (7.69)	52.59 (7.31)	47.50 (5.52)	**6.00*** **	NO < ID / AT+
Hopelessness [e,f]	8.82 (4.64)	14.48 (4.38)	14.29 (4.33)	7.33 (5.07)	**23.93*** **	NO < ID / AT+<br>AT- < ID / AT+
Resilience[e, h]	46.28 (14.78)	34.07 (14.25)	36.57 (12.95)	49.18 (10.77)	**9.98*** **	NO > ID /AT+<br>AT- > ID
Anxiety (BAI)[e, g]	16.39 (11.57)	23.17 (11.49)	26.80 (13.20)	14.92 (10.52)	**6.07*** **	NO < ID / AT+<br>AT- < AT+
**Childhood maltreatment**						
CTQ total score[d]	37.58 (9.58)	42.96 (13.81)	44.53 (14.17)	44.38 (24.33)	**2.89*[j]**	NO < AT+
Number of abuse types[c]	0.61 (.96)	1.02 (1.33)	1.32 (1.49)	1.00 (1.77)	6.34[c]	
**NSSI**						
NSSI in past year	32 (43.2%)	70 (70%)	51 (91.1%)	9 (75%)	**34.29*** **	NO < ID / AT+<br>ID < AT+
Self-harm frequency[b]	1.41 (2.23)	3.98 (3.30)	5.89 (3.75)	2.00 (2.63)	**56.65***[c]**	NO < ID / AT+<br>ID < AT+<br>AT- < ID / AT+
Number of methods	0.75 (1.02)	1.65 (1.13)	2.47 (1.25)	1.17 (1.27)	**52.91***[c]**	NO < ID / AT+<br>ID < AT+<br>AT- < AT+

self-battering	15 (23.8%)	62 (63.3%)	41 (77.4%)	4 (33.3%)	**40.21*** **	NO < ID / AT+<br>AT- < AT+
burning	6 (9.5%)	15 (15.5%)	18 (34.0%)	1 (8.3%)	**13.52** **	NO < AT+
cutting (deep)	6 (9.5%)	21 (21.4%)	29 (54.7%)	4 (33.3%)	**32.53*** **	NO / ID < AT+
cutting / scratching	20 (31.7%)	62 (63.3%)	43 (81.1%)	5 (41.7%)	**31.78*** **	NO < ID / AT+<br>AT- < AT+
**Substance use**[i]						
smoking (tried once)	33 (44.6%)	56 (54.9%)	42 (79.2%)	9 (75%)	**15.55*** **	NO / ID < AT+
alcohol (tried once)	41 (55.4%)	78 (76.5%)	44 (83.0%)	9 (75%)	**13.99** **	NO > ID / AT+
cannabis (tried once)	23 (31.1%)	49 (48.0%)	27 (50.9%)	8 (66.7%)	**9.19***	nonsignificant
lifetime substance abuse score [i]	1.49 (1.68)	2.11 (1.65)	2.77 (1.90)	2.58 (1.98)	**18.12*****[c]	NO < ID / AT+<br>NO < AT-
global continuum of substance risk score[i]	10.93 (15.74)	16.70 (20.58)	24.02 (23.36)	27.75 (30.76)	**5.03****[b]	NO < AT-

Note: Statistics are Chi-square for categorical and one-way ANOVA for continuous variables. *p < .05; **p <.01;*** p < .001; [a]controlled for gender; [b]WelchTest, [C]Kruskal-Wallis, [d]35 missing, [e]controlled for depression severity, [f]13 missing, [g]1 missing, [h] 3 missing, [i] 5 missing, [j] transformed, CDRS-S = Children's Depression Rating Scale minus the two items related to suicidal / morbid ideation.

SI Table 5: Regression coefficients, standard errors, and model summary information for the influence of childhood trauma on suicidal ideation based on a serial multiple mediator model including hopelessness and depression severity

		**BHS**				**CDRS-S**				**SIQ**		
		Coeff.	SE	p		Coeff	SE	p		Coeff.	SE	p
**CTQ**	a_1	0.095	0.024	< .001	a_2	0.079	0.037	0.036	c'	0.045	0.100	0.657
**BHS**					d_{21}	0.449	0.096	< .001	b_1	2.033	0.271	< .001
**CDRS-S**									b_2	0.549	0.191	0.005
**Gender**		1.289	0.982	0.191		2.230	1.188	0.062		1.908	3.091	0.538
		R^2 = 0.077				R^2 = 0.171				R^2 = 0.337		
		F (2,195) = 9.445, p < .001				F (3,194) = 15.975, p < .001				F (4,193) = 29.445, p < .001		

Note: CTQ = Childhood Trauma Questionnaire; BHS = Beck Hopelessness Scale; CDRS-S = Children's Depression Rating Scale minus the two suicide-related items; SIQ = Suicidal Ideation Questionnaire, Coeff = standardized coefficient, SE = standard error.
